# Laboratory tests for controlling poultry red mites (*Dermanyssus gallinae*) with predatory mites in small ‘laying hen’ cages

**DOI:** 10.1007/s10493-012-9596-z

**Published:** 2012-07-08

**Authors:** Izabela Lesna, Maurice W. Sabelis, Thea G. C. M. van Niekerk, Jan Komdeur

**Affiliations:** 1 Section Population Biology, Institute for Biodiversity and Ecosystem Dynamics, University of Amsterdam, Science Park 904, 1098 XH Amsterdam, The Netherlands; 2 Animal Ecology Group, Centre for Ecological and Evolutionary Studies, University of Groningen, Nijenborgh 7, 9747 AG Groningen, The Netherlands; 3 Livestock Research, Edelhertweg 15, 8219 PH Lelystad, The Netherlands

**Keywords:** Biological control, Ectoparasite, Poultry red mite, *Dermanyssus gallinae*, Predatory mites, *Androlaelaps casalis*, *Stratiolaelaps scimitus*, Laying hen, *Gallus gallus*

## Abstract

To assess their potential to control poultry red mites (*Dermanyssus gallinae*), we tested selected predaceous mites (*Androlaelaps casalis* and *Stratiolaelaps scimitus*) that occur naturally in wild bird nests or sometimes spontaneously invade poultry houses. This was done under laboratory conditions in cages, each with 2–3 laying hens, initially 300 poultry red mites and later the release of 1,000 predators. These small-scale tests were designed to prevent mite escape from the cages and they were carried out in three replicates at each of three temperature regimes: 26, 30 (constant day and night) and 33–25 °C (day-night cycle). After 6 weeks total population sizes of poultry red mites and predatory mites were assessed. For the temperature regimes of 26 and 33/25 °C *S. scimitus* reduced the poultry red mite population relative to the control experiments by a factor 3 and 30, respectively, and *A. casalis* by a factor of 18 and 55, respectively. At 30 °C the predators had less effect on red mites, with a reduction of 1.3-fold for *S. scimitus* and 5.6-fold for *A. casalis*. This possibly reflected hen manure condition or an effect of other invertebrates in the hen feed. Poultry red mite control was not negatively affected by temperatures as high as 33 °C and was always better in trials with *A. casalis* than in those with *S. scimitus*. In none of the experiments predators managed to eradicate the population of poultry red mites. This may be due to a prey refuge effect since most predatory mites were found in and around the manure tray at the bottom of the cage, whereas most poultry red mites were found higher up in the cage (i.e. on the walls, the cover, the perch, the nest box and the food box). The efficacy of applying predatory mites in the poultry industry may be promoted by reducing this refuge effect, boosting predatory mite populations using alternative prey and prolonged predator release devices. Biocontrol success, however, will strongly depend on how the poultry is housed in practice (free range, cage or aviary systems) and on which chemicals are applied to disinfect poultry houses and to control other pests.

**Electronic supplementary material:**

The online version of this article (doi:10.1007/s10493-012-9596-z) contains supplementary material, which is available to authorized users.

## Introduction

While there is progress in exploring pathogens (mainly fungi) as a means to combat ectoparasites on livestock (Hogsette 1999; Wall 2007), biological control by means of true predators is still in its infancy. Which predators are suitable for this purpose, critically depends on the extent to which the ectoparasites reside off- and on-host and on the extent to which the predators match the parasite distribution. In this respect, the poultry red mite (PRM), *Dermanyssus gallinae* (De Geer) (Acari: Dermanyssidae), a world-wide pest of chickens (Axtell and Arends 1990; Sparagano 2009), represents an interesting case. Under natural conditions PRM is a blood-sucking ectoparasite in nests of birds and small mammals and visits its host only during night (Wood 1917). During the day it stays off-host and hides in the direct environment of its host, i.e. the nest material and its immediate surroundings. Nests usually harbour communities of arthropods, including not only parasites, but also microbivores and predators that feed on them (Fenda 2010; Lesna et al. 2009 and references therein). Such communities may harbour several species that are specialized on life in a nest (nidicoles), and they emerge more easily in nests that are re-used by the nesting bird or mammal. This is why we made inventories of arthropods living in starling nests and carried out experiments to assess which of them are true predators of PRM (Lesna et al. 2009). This yielded several candidate predators for biological control of PRM, one of which was particularly abundant (especially in re-used nestboxes), shown to be a true predator of PRM (see also McKinley 1963) and demonstrated *not* to be a facultative parasite of (young) birds: *Androlaelaps casalis* (Berlese).

This predator was also found spontaneously in some Dutch poultry houses harbouring PRM as a pest of laying hens (Lesna et al. 2009). Whereas other laelapid predators known to feed on PRM, i.e. *Hypoaspis aculeifer* (Canestrini) and *Stratiolaelaps scimitus* (Womersley) (Walter and Campbell 2003), were rarely ever found in starling nests nor in Dutch poultry houses in 2007–2009 (Lesna et al. 2009), *S. scimitus* was found to be abundant in PRM-infested backyard poultry houses of small farmers in Minas Gerais, Brazil (Lesna, pers. obs. November 2008). Given that this predator feeds on PRM (Tuovinen 2008) and is currently used to control *D. gallinae* and other blood feeding mites on pet animals, such as canaries, pigeons and reptiles (John Evers, REFONA, pers. comm. 2008), *S. scimitus* represents another true predator for biological control of PRM. For these reasons, we selected *A. casalis* and *S. scimitus* as candidate predators for PRM control and carried out experiments to test their efficacy in reducing PRM populations.

To demonstrate the impact of predatory mites on PRM it is wise not to take a big leap from laboratory tests on predation (Lesna et al. 2009) to biocontrol tests in poultry houses. This is because poultry management in practice involves various measures that may interfere with biological control, thereby hampering interpretation of biocontrol tests. Among the measures taken are the use of pesticides, such as pyrethroids (Marangi et al. 2009) and organo-phosphates (Roy et al. 2009), the use of disinfectants, such as ethoxyquin and formalin, and the use of inert materials with mainly physical effects, such as oils (diesel, petroleum) and silicas (Maurer et al. 2009; Kilpinen and Steenberg 2009). These chemicals or their residues may have an impact on predatory mites, but the extent to which they interfere with biological control is still to be assessed. Another good reason not to proceed immediately to practice is that commercially kept laying hens are accommodated in widely different housing systems, such as free-range, cage and aviary systems, which do not only differ in structure but among others also in manure management. Last, but not least, it is not feasible to carry out proper control experiments in commercial poultry houses.

For all these reasons it is better to do biocontrol experiments first in simplified environments under conditions where no pesticides, disinfectants, inert dusts or oils are used. Moreover, it is necessary to regulate temperature in order to assess whether high temperatures can negatively affect PRM biocontrol. In poultry houses, periods of hot weather can lead to high temperatures up to 33 °C (Nico Harteveld, pers. comm. 2009). Such high temperatures can negatively affect survival of laelapid predators (Lesna et al. 2009). In this article, we therefore report on the results of biocontrol tests with *A. casalis* and *S. scimitus* at three temperature regimes (26 °C, 30 °C and a diurnal cycle between 33 °C during the day and 25 °C at night), and in small cages specially designed to accommodate a few laying hens and equipped with the most relevant facilities in poultry houses, such as a perch, a nest box, a feed box, a water source, a floor with litter and a manure deposit.

## Materials and methods

### Origin of the mites released

PRMs were collected in 2009 from poultry houses at the Experimental Farm of Wageningen University—Livestock Research in Lelystad, The Netherlands. The predatory mite *A. casalis* was originally collected in March 2007 from nests of European starlings at Vosbergen Estate (Eelde-Paterswolde, The Netherlands) (Lesna et al. 2009), first maintained in the laboratory on a diet of *Acarus siro* L. and then mass-reared in 2009 by Koppert Biological Systems (Berkel en Rodenrijs, The Netherlands). The predatory mite *S. scimitus* was already mass-reared for many years by Koppert Biological Systems. A precise description of the original sampling site is lacking and we have never found this species from starling nests nor from Dutch poultry houses (Lesna et al. 2009). In November 2008, however, we found *S. scimitus* in association with PRM in backyard poultry houses of small farmers near Viçosa in Brazil (Lesna and Sabelis, pers. obs.; identification validated by Dr Farid Faraji). This strain had the same predation rate as the one mass-reared by Koppert Biological Systems (Lesna, pers. obs.) and this is one reason why we selected the latter strain of *S. scimitus* for the biocontrol experiments described in this article. The other reason was that this strain is already used for several years to control PRM and other blood feeding mites on pet animals, such as canaries, pigeons and reptiles (John Evers, REFONA, pers. comm. 2008).

### Design of the laying hen cages

**Fig. 1:**
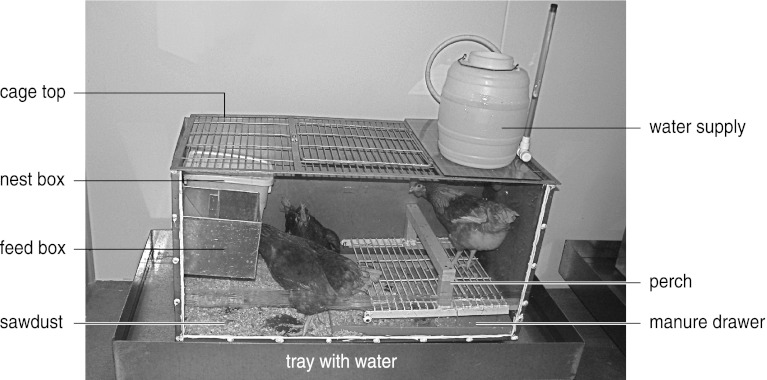
Design of the poultry cage used to study the dynamics of PRM and predatory mites. The metal cage has an area with litter (pine wood shavings) and one with a manure drawer at the bottom, a metal grid with an entrance and a plastic water tank (*right-side*) on top, a transparant Perspex wall at the front, a wall with a metal feed box and a plastic nest box at the *left side* and a wooden perch above the manure drawer at the bottom. The cage contained two or three laying hens

The biocontrol experiments were carried out in 12 metal cages (1.5 *×* 0.7 *×* 0.7 m) with a plexiglass front and a metal grid as a roof (Fig. 1). Each cage housed three beak-trimmed laying hens (race Lohmann Brown) in the first trial and two of such hens in the consecutive trials. The cages had a white-plastic water tank on the roof, a metal feed-box halfway the left wall, a white plastic box for egg laying next to the feed-box, a manure drawer at the bottom under a metal grid serving as a floor and a wooden perch 20 cm above the manure drawer (Fig. 1). The manure drawer covered approximately 50 % of the cage floor, whereas the remainder was filled with pine wood shavings. Water was continuously accessible to the hens through a nipple drinker connected to the water tank. On a daily basis, the employees of Livestock Research provided commercially available layer feed and they also collected the eggs laid by the hens. Manure was removed once per week and a new layer of wood shavings was provided when necessary. Employees of Livestock Research had the task to observe any aggressive interactions between hens occupying the same cage. In the first series of experiments at 26 ± 1 °C (60 ± 5 % RH), there were three laying hens per cage and one replicate, a cage under treatment with *S. scimitus*, had to be terminated due to escalation of aggressive behaviour among the hens. In subsequent series at higher temperatures, we introduced only two laying hens per cage and did not have to terminate any replicates for reasons of aggressive behaviour of the hens. Preliminary experiments showed that maximum growth rates of PRM are achieved independent of whether 1, 2 or 3 laying hens were provided. Evidently, blood resources are not limiting given the number of PRM introduced and the period under consideration (see below). For reasons of animal welfare it was important to test this a priori to avoid exposing the hens to an overload of ectoparasites.

### Biocontrol experiments

Cages and laying hens were ensured to be PRM-free and the cages were placed on bricks in a large tray with a layer of water reaching just below the bottom of the cage and serving as a barrier to mite migration. One or two days after introducing the laying hens into the cages, each cage received a Petri dish with 300 PRM (only mobile stages) that was placed in the manure drawer under the grid covering the drawer (to prevent the hens from picking at the dish). The lid of the Petri dish was removed to allow PRMs moving into the cage.

The 12 cages prepared as above were divided equally over three treatments: (1) predator-free control (replicate 1–4) and predator treatment with either (2) *S. scimitus* (replicate 5–8) or (3) *A. casalis* (replicate 9–12). Due to limitations of space and the number of cages three series of experiments with 12 cages each were carried consecutively. The first series of experiments was carried out at 26 ± 1 °C (60 ± 5 % RH) (March–April 2009), the second series at 30 ± 1 °C (55 ± 5 % RH) (May–July 2009) and the third (and last) series at 33 ± 1 °C during the day but—as a compromise with animal welfare considerations—25 ± 1 °C during the night (55 ± 5 % RH) (August–September 2009). In all experiments TL light sources were switched on at 4:00 AM and switched off at 8:00 PM (16:8 LD cycle).

In the first series the predators were released 10 days after PRM introduction, but in the other two series they were released earlier, i.e. 3 days after PRM introduction, to give PRM less of a head start. In all predator treatments c. 1,000 predatory mites were released following the same method as explained for PRM introduction (except that plastic vials were used instead of Petri dishes). Each of the three series of experiments lasted 7 weeks since PRM introduction (note that the 7 weeks were counted from 0 to 6). At the last day of each experiment first the laying hens were removed from the cages by employees of Livestock Research. These hens were not inspected for the presence of ectoparasites because they were taken away during the day when most of the PRM are off-host. Subsequently, we collected the mites from different parts of each cage (roof, water-tank, walls of the feed-box, laying nest, walls, perch) by brushing the surfaces (broad brushes of the elma 59-1 type) and thereby the mites thereon into vials with 70 % alcohol. The content of the alcohol vials was poured into large Petri dishes with sections drawn on the bottom of the dish. After stirring the solution to obtain a homogeneous distribution of mites in the alcohol layer, we used a binocular microscope to count the number of mites (PRM, *S. scimitus* and *A. casalis*) in a section equivalent to 1/8 of the solution. In addition, a fraction (c. 3 %) of the manure and litter on the bottom of the cage was put into small plastic bags and then after spreading the material on white paper the mites were counted and collected by the aid of a fine brush and transferred into vials with 70 % alcohol. In this article we only show the total numbers of mites as obtained by linear extrapolation from the fractions. Thus, the main result obtained for each cage in each of the three series of experiments consisted of the total number of PRM and the total number of predatory mites after 7 weeks since the introduction of 300 PRM.

### Statistical analysis

Since our experiments usually yielded 3 or more successful replicates per treatment for each temperature level, it was possible to perform statistical analyses. We decided to apply parameter-free methods as these are more conservative with respect to the null hypothesis and therefore critically test whether the release of predatory mites has an impact on PRM numbers. This is why we ranked the total numbers of PRM per cage in week 6 and subjected the ranked data to a Kruskal–Wallis test for differences in location (Sokal and Rohlf 1995). If this test was significant (*P* ≤ 0.05) or bordering significance, it was followed by a Wilcoxon two-sample test to detect significant differences between treatments (Sokal and Rohlf 1995). None of the ranked data sets for parameter-free tests (Kruskal–Wallis and Wilcoxon two-sample tests) had ties, making corrections for ties obsolete.

To avoid confounding with temperature (and temperature-related factors), these tests were carried out for each temperature regime (Table 1). Subsequently, we applied the same parameter-free test procedure to detect an effect of temperature on (1) PRM densities in week 6 within the series of control experiments, (2) PRM densities in week 6 within the two series of predator treatments taken together and (3) predator densities in week 6 within the two series of predator treatments taken together (Table 2). Finally, we compared the distributions of the two predator species by ranking the fractions of their total populations residing on the bottom of the cage and then by applying a Wilcoxon two-sample test to detect a difference in location of the ranked data.

**Table 1:** Kruskal–Wallis tests and Wilcoxon two-sample tests to detect effects of predator treatments on reduction of final PRM densities (measured in week 6) (C = control treatment; S = treatment with *Stratiolaelaps scimitus*; A = treatment with *Androlaelaps casalis*) at each of three temperature regimes

Temperature	N	Kruskal–Wallis test-statistic (*P* value)	Wilcoxon two-sample test statistic (*P* value)		
Regime			C vs. S	C vs. A	S vs. A
26 °C	10	7.76 (0.021*)	11 (0.10)	9 (0.05*)	7 (>0.10)
30 °C	8	1.80 (0.41)	–	–	–
33/25 °C	11	7.84 (0.019*)	12 (0.05*)	16 (0.025*)	10 (>0.10)

Test-statistics are provided and in parentheses the *P* value is given for which χ_[*df*=3−1=2]_^2^ approximates the test statistic (*df* = degrees of freedom)

* Significance; N the number of successful experiments per temperature regime

**Table 2:** Kruskal-Wallis tests and Wilcoxon two-sample tests to detect effects of temperature regime (26, 30 or 33/25 °C) on final PRM densities in the control treatment, final PRM densities in the two predator treatments taken together and final predator densities in the two predator treatments taken together

Variable	Kruskal-Wallis test-statistic (*P* value)	Wilcoxon two-sample test statistic (*P* value)		
		26 vs. 30 °C	26 vs. 33/25 °C	30 vs. 33–25 °C
PRM in control	5.00 (0.082)	6 (>0.10)	12 (0.05*)	10 (>0.10)
PRM in treatments	12.41 (0.002*)	23 (>0.10)	49 (<0.001*)	32 (0.005*)
Predators in treatments	4.07 (0.13)			

Test-statistics are provided and in parentheses the *P* value is given for which χ_[*df*=3−1=2]_^2^ approximates the test statistic (*df* degrees of freedom). * Significance

## Results

### Biocontrol experiments

Not all mite introductions into the cages were successful. If the mites in the samples (from Nordenfors traps for PRM and from manure for predatory mites) showed no numerical increase in the first 3 weeks and the estimated final number in week 6 was close to zero, they were considered to have failed in establishing a population. Out of the total of 36 replicates from three series of population experiments, each at a different temperature and each in 12 replicates, the introduced populations of PRM and those of the predatory mites were successfully established in 29 replicates (c. 80 %). The reasons for replicate failure varied. At 26 °C one replicate (nr 9) had to be stopped due to aggressive pecking behaviour of the laying hens and another replicate (nr 4) failed due to inadvertent invasion of *A. casalis*. At 30 °C four replicates (nr 4, 5, 6 and 9) failed due to invasions of *Macrocheles* species, possibly via the feed provided to the hens. The populations of *Macrocheles* species had reached such large numbers that it was impossible to determine which predator exerted the main effect and this is why these four replicates were discarded from further analysis. At 33–25 °C only one replicate (nr 6) was discarded due to failure of population establishment of the introduced species. Thus, for statistical analysis of the control, the treatment with *S. scimitus* and the treatment with *A. casalis*, we had respectively 3, 4 and 3 replicates for the 26 °C series, 3, 2 and 3 replicates for the 30 °C series and 4, 3 and 4 replicates for the 33–25 °C series. All these replicates shared the property of showing an increase of the PRM population in the Nordenfors samples taken during at least the first 3 weeks, thus confirming PRM establishment after introduction to the cage (see supplementary material). Moreover, the manure/litter samples from the replicates subject to predator treatments showed all evidence for establishment of the predator species introduced (see supplementary material).

**Fig. 2:**
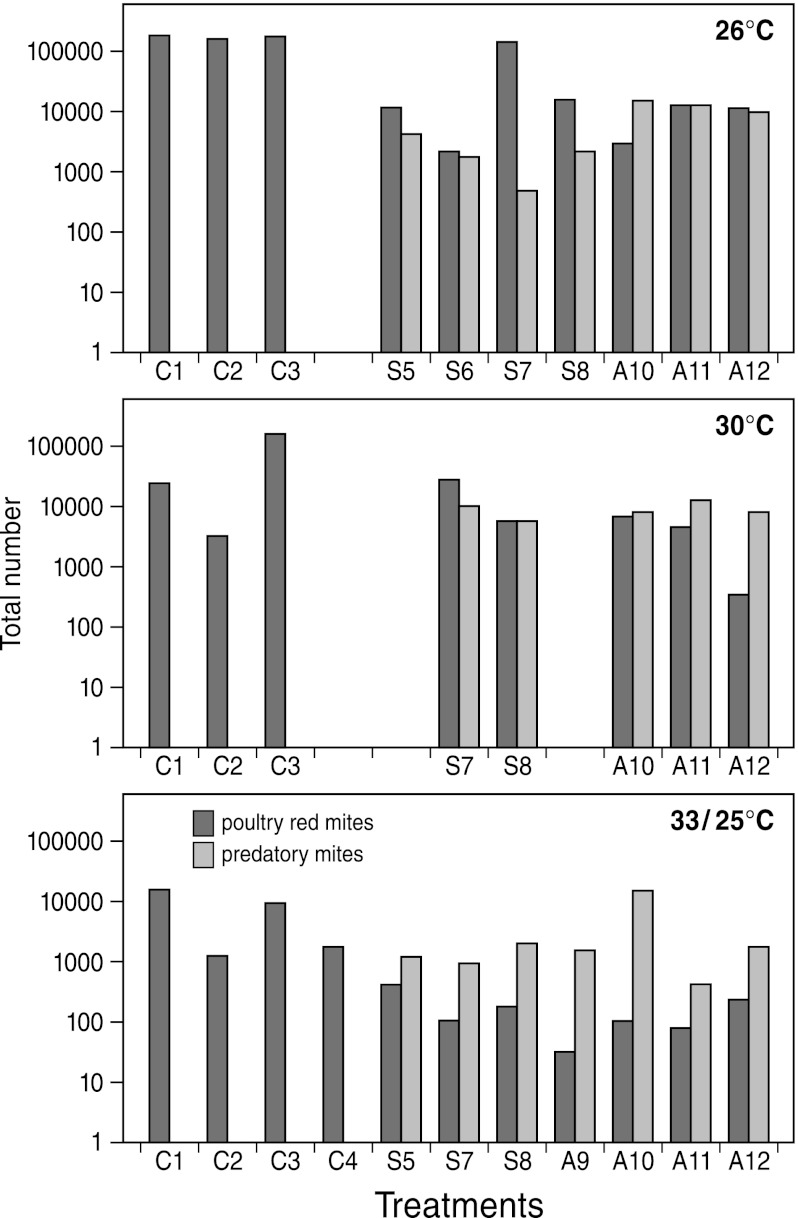
Total numbers of poultry red mites (*black*) and predatory mites (*white*) estimated from the final (week 6) assessment carried out at 26, 30 and 33/25 °C and treatments without predators (C), with the predatory mite *Stratiolaelaps scimitus* (S) and with the predatory mite *Androlaelaps casalis* (A) (4 replicate experiments per treatment). The cage number is given next to the treatment symbol (C, S, A). Missing numbers represent cages in which the replicate experiment failed for reasons explained in the text

Based on the replicates showing evidence for successful establishment we performed statistical analyses to assess whether the predatory mites significantly reduced final PRM densities (in week 6) beyond those in the control (data shown in Fig. 2). First, we carried out a Kruskal–Wallis test for each temperature series separately and found significant differences in the location of the ranked data at 26 °C and 33/25 °C, but not at 30 °C (Table 1). Then, we applied Wilcoxon two-sample tests on ranked data (Table 1) and found that the treatment with *A. casalis* differed significantly from the control treatment at 26 °C as well as at 33/25 °C, that the treatment with *S. scimitus* differed significantly from the control treatment at 33/25 °C, but not at 26 °C, and that in none of the temperature series the two predator treatments differed significantly from each other. Strikingly, however, the treatments with *A. casalis*, both at 26 and 33/25 °C, differed more significantly from the control than the treatment with *S. scimitus* (Table 1). This trend was also found when calculating the factor expressing reduction of PRM numbers relative to those in the control treatment (Fig. 2). At 26 °C *S. scimitus* reduced PRMs by a factor 3.7 (from 168,163 in the control to 45,270 in treatment), whereas *A. casalis* reduced them by a factor 18 (from 168,163 in the control to 9,356 in the treatment). At 33/25 °C *S. scimitus* reduced PRMs by a factor 29 (from 7,339 in the control to 250 in the treatment), whereas *A. casalis* reduced them by a factor 55 (from 7,339 in the control to 133 in the treatment). Also at 30 °C, *A. casalis* reduced PRMs 4.2 times more than *S. scimitus* (from 68,749 in the control to 17,795 in the *S. scimitus* treatment and to 4,196 in the *A. casalis* treatment).

The experiments were carried out at three temperature regimes to assess whether high temperatures recorded in poultry houses (Nico Harteveld, pers. comm. 2009) could hamper biological control. However, our experiments provided no reason to suspect such a negative effect because excellent population establishment (only one replicate failed) and the most severe PRM reduction was obtained in the series at 33/25 °C (Fig. 2). A negative effect at 30 °C may be inferred from the non-significant treatment effects and the low PRM reduction (1.3 for *S. scimitus* and 5.6 for *A. casalis* at 30 °C), but this may not be realistic because there were large numbers of alternative prey, mainly acarids (>10,000), nematodes (uncountable) and fly larvae (>1,000). The acarids were found to originate from the chicken feed used for the 30 °C experiments and the same applied to the many macrochelid mites (and to relatively fewer cheyletid mites) which in some replicates even reached such large numbers that they had to be discarded from further analysis. The abundance of nematodes and fly larvae was probably related to the liquid state of the chicken manure and hence the condition of the laying hens. The presence of these alternative prey may initially have reduced predation on PRM.

In the control experiments the overall numbers of PRM in week 6 were largest at 26 °C (Fig. 2), corresponding to population growth rates (*r*) in each of the 4 replicate controls (0.152, 0.147, 0.151 females/female/day for exponential growth with *N*(0) = 300 and *t* = 42 days) that are somewhat higher than reported by Maurer and Baumgärtner (1992) (i.e. 0.12 females/female/day based on life table analysis at 25 °C). However, the population growth rate in each of 3 replicate control experiments at 30 °C (0.105, 0.055, 0.152 females/female/day) and especially those in each of the 4 replicate control experiments at 33/25 °C (0.094, 0.033, 0.085, 0.048 females/female/day) were lower (Fig. 2), which was striking enough to put this to a statistical test (Table 2). A Kruskal–Wallis test revealed a difference bordering significance (*P* = 0.082) with respect to location of the ranked data when compared to those of all three control experiments. A subsequent Wilcoxon two-sample test showed a significant difference between the experiments at 33/25 °C and those at 26 °C, whereas those at 30 °C were not significantly different from either of the two (Table 2). Considering relative PRM numbers in the control experiments in week 6, those at 26 °C were 2.4 times higher than those at 30 °C and 22.9 times higher than those at 33/25 °C (Fig. 2). This indicates a negative effect of high temperatures on the population growth of PRM.

Another striking feature is that—taken together– the two predator treatments at 26 °C had higher PRM numbers in week 6 (on average 29,878 PRMs/cage) than those at 30 °C (on average 9,636 PRMs/cage) and 33/25 °C (on average 183 PRMs/cage). A Kruskal–Wallis test revealed a significant difference in location of the ranked data from all experiments involving predator treatments at all three temperature regimes (Table 2) and subsequent Wilcoxon two-sample tests showed a significant difference between PRMs at 33/25 °C and 26 °C, whereas those at 30 °C were not significantly different from those at 26 °C, yet significantly different from those at 33/25 °C (Table 2). Among the factors that may contribute to this trend, one could think of (1) the negative effect of temperature on PRM population growth (as inferred above), (2) the later introduction of predators in the experiments at 26 °C (12 instead of 3 days after PRM release; see M&M) and (3) a temperature effect on predator population growth. However, the latter possibility was not supported by a Kruskal–Wallis test (Table 2). Predator densities in week 6 were relatively the highest at 30 °C (an average of 9,450 predators/cage versus 6,550 at 26 °C and 3,466 at 33–25 °C). Because alternative prey (acarids, fly larvae) was very abundant only in the treatment at 30 °C, the higher predator densities may be due to larger supply of prey. In addition, predator densities at 26 °C were higher than those at 33/25 °C, which may also be due to a larger prey supply: PRM densities at 26 °C were much higher than those at 33/25 °C (for reasons given above).

### Mite distribution in the biocontrol experiments

**Fig. 3:**
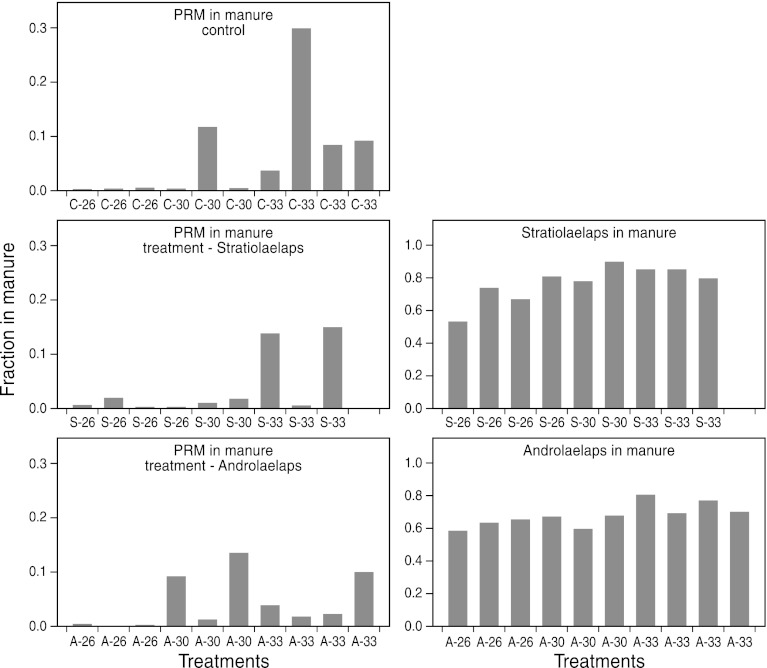
Distribution of poultry red mites (PRM) (*left*) and predatory mites (*Stratiolaelaps scimitus* and *Androlaelaps casalis*) (*right*). Shown is the fraction of the total population present at the bottom of the cage (i.e. in and around the manure tray) at 26, 30 and 33–25 °C and for treatments without predators (*top*), with the predatory mite *A. casalis* (A) (*middle*) and with the predatory mite *S. scimitus* (S) (*bottom*). Note the difference in scale between left-hand and right-hand panel

The biocontrol experiments also provided information on how PRM and its predators were distributed within the cages during the final assessments in week 6. Note that these assessments were done during the day, which is important because PRMs exhibit diurnal foraging behaviour (off-host during the day and, when motivated to acquire blood, on-host during night) and we cannot exclude diurnal rhythms in foraging of the predatory mites. In general, PRMs were found higher up in the cage: along the cage walls, on the cage roof (including the water tank), under the roofs of the feed-box and the laying nest and underneath the perch. The predators, however, were concentrated at the bottom of the cage: in the manure and litter under, in and around the manure tray. To quantify the extent to which this differential predator–prey distribution was manifested, we calculated the fractions of the total populations residing at the bottom of the cage, further referred to as ‘fraction in manure’ because the majority of mites at the cage bottom were found under, in and around the manure tray (Fig. 3). It does not require a statistical test to see that most predatory mites are at the bottom of the cage, whereas most PRM are away from the bottom and thus higher up in the cage. To test whether the PRM distribution is affected by temperature, we ranked the fractions of PRMs in the manure of the control treatments and applied a Kruskal–Wallis test showing that there was no significant difference in location of the ranked data (*P* = 0.114). Wilcoxon two-sample tests further showed that relatively more PRMs reside at the bottom of the cage at 33/25 °C than at 26 °C (*P* = 0.05), but PRM fractions at 30 °C did not differ significantly from those at any of the other temperature regimes. We also tested whether the PRM distribution was affected by the presence of predators. To avoid confounding with temperature we tested the ranked data per temperature regime. The Kruskal–Wallis tests, however, did not reveal any significant effects of predator presence in any of the temperature regimes.

Finally, we tested whether the distribution of *S. scimitus* differed from that of *A. casalis*. As there were no significant effects of temperature on these distributions (Kruskal–Wallis tests), we ranked the fractions from all temperature regimes and applied a Wilcoxon two-sample test (i.e. 9 fractions for *S. scimitus* and 10 for *A. casalis*), which showed a significant difference between the distributions of the two predator species (Wilcoxon test statistic = 70; *P* = 0.025; Fig. 3). When considering the mean fractions over all replicates for each of the two predator species, it can be seen that 77.2 % of the *S. scim*itus population resides in the manure as opposed to 68.3 % of the *A. casalis* population (Fig. 3). We conclude that both predator species have populations concentrated in the manure, but *A. casalis* does so to a lesser extent than *S. scimitus*.

## Discussion

Because current methods to control PRM in Dutch poultry farms are not sufficiently effective (Emous et al. 2005), we aimed to develop new methods to combat poultry red mites by the use of their natural enemies, in particular predatory mites. This approach was pioneered by Buffoni et al. (1995, 1997) and Maurer and Hertzberg (2001). They reported spontaneous occurrence of the predatory mite *Cheyletus eruditus* (Schrank) in the litter of poultry houses in Switzerland (Maurer et al. 1993) and observed this mite feeding on juvenile PRMs, but did not observe sufficient PRM control in their experiments in poultry houses (Maurer and Hertzberg 2001). We pursued another approach to develop biological control methods by identifying predators of poultry red mites in their natural habitat. This is why we analysed the food web structure of arthropods in PRM-infested bird nests, as well as in commercial and non-commercial poultry houses. This yielded two candidate predatory mites: *A. casalis* from nests of the European starling (Lesna et al. 2009) and *S. scimitus* from backyard poultry houses in Brazil (Lesna, pers. obs. 2008). The biocontrol tests described in this article showed that in small cages with a few laying hens the two predator species reduced PRM populations relative to the control, but the impact of the two predator species did not statistically differ from each other. However, quantitatively, reduction of PRM at all three temperature regimes was stronger in trials with *A. casalis* compared to trials with *S. scimitus*. Temperatures as high as 33 °C reduced the population growth of PRM, but had no negative effect on the extent to which PRM was reduced by the predatory mites.

Apart from the impact of predatory mites on PRM one of the most striking results was that predators and PRMs are differentially distributed within cages. Whereas most PRMs were found higher up in the cage structure (roofs of the feed box, the nest box and the cage including the water tank), the predatory mites were mainly found on the cage floor (under, in and around the manure drawer). As expected, PRMs were also found in the Nordenfors trap fixed under the perch. As sampling was performed during the day and PRMs exhibit a diurnal rhythm in being off- and on-host (Wood 1917), we expect that most PRMs have to pass the bottom of the cage to reach and parasitize the laying hens while they are roosting on the perch during night. This may be a crucial feature of the cage we used, but this may not apply to poultry farms, especially those with cage and aviary systems. Thus, to improve biocontrol under these practical conditions it is mandatory to create artificial environments in which predatory mites can thrive and which can be positioned higher up in the structural elements of cage and aviary systems. Moreover, it is important to use predator species that are arrested less on the floor and in the manure. In this respect it is interesting to note that in our cage experiments *A. casalis* was significantly less concentrated on the bottom of the cage than *S. scimitus*.

As suggested by some of our experiments, overall prey supply, including prey other than PRM, may play a role in boosting populations of predatory mites and sooner or later this may backfire on the PRM populations (Van Rijn et al. 2002). Both *A. casalis* and *S. scimitus* are capable of feeding and reproducing on a diet of acarids as prey. This was in fact crucial to establish mass cultures of either of the two predator species. Thus, it is worthwhile to investigate the possibilities of providing alternative prey (e.g. acarid mites; see Lesna et al. 2009) to promote predators and suppress PRM. We suggest to enrich the artificial environments discussed above with alternative prey for the predators, thereby creating slow-release units reminiscent of those used for the release of phytoseiid predatory mites in greenhouse crops.

The results presented in this article represent no more than a first step in showing the efficacy of predatory mites in controlling PRM. Although the experimental conditions in small cages are close to those in practice, they can never exactly match in terms of scale, housing systems (free-range, cage or aviary) used in the poultry industry and in terms of chemicals applied to control pests and disinfect poultry houses. The next step will be to critically test the effectiveness of biological control under practical conditions. Application in practice, however, has bypassed such a test, as the predatory mites are commercially available since 2010 for red mite control in the poultry industry (Companies PROTEKTA and REFONA, The Netherlands).

## Electronic supplementary material

Below is the link to the electronic supplementary material.

Supplementary material 1 (DOCX 43 kb)

Supplementary material 2 (PDF 44 kb)

Supplementary material 3 (PDF 70 kb)
