# Supplementary material for: Laboratory tests for controlling poultry red mites (Dermanyssus gallinae) with predatory mites in small ‘laying hen’ cages
Source: Exp Appl Acarol. 2012 Jul 8;58(4):371–83. doi: 10.1007/s10493-012-9596-z (PMC3487000; doi:10.1007/s10493-012-9596-z)
Supplement: Supplementary file 1 — Supplementary material 1 (DOCX 43 kb) [file 10493_2012_9596_MOESM1_ESM.docx]

**Supplementary material**

*Weekly sampling*

Apart from the assessments of total numbers at the end of the experiment (week 6) we also performed weekly sampling trials to monitor the population trends (Figure S1). According to Zenner et al. (2009) it is more efficient to place cardboard traps for PRM sampling near sites frequented by the laying hens and this is why they advocated placing the traps on the perches rather than in the nest-box. We used a small version of the so-called Nordenfors trap (15 cm^2^ pieces of corrugated cardboard; Nordenfors and Chirico, 2001) that fitted under the wooden perch because there the traps are less likely to be pecked at or even removed by the hens. Since predatory mites are rarely collected in Nordenfors traps (Lesna, pers. obs.), we also took samples from the litter and the manure (c. 3% of the total material) to monitor predatory mites and PRM. Each sample represents c. 3.3% (1/30) from the total amount of manure/litter.

*Population trends*

Sampling once per week was necessary to determine whether the introduced mite populations (PRM and predators) had established or not, but it was also done to see whether qualitative changes in population size can be identified (Figure S2). As a qualitative measure of the trend in population size we scored how frequent mite density in a sample from week *N* in a given replicate cage was followed by a larger mite density in a sample from week *N*+*1* in the same replicate cage. For PRM density in the control experiments (sampled weekly by Nordenfors traps under the perch) we found 16 scores of larger mite density in 16 cases at 26ºC, 10 such scores out of 15 cases at 30ºC and 20 such scores out of 20 cases at 33/25ºC (Figure S1), making a grand total of 46 scores out of 51 cases (90.2%). As we would expect consistently growing PRM populations in the control experiments (Kirkwood, 1963; Maurer and Baumgartner, 1992, 1994; Buffoni et al., 1995, 1997) these scores show that the sample method predicts the trend right in at least 90% of the times. For PRM density in the predator treatments we have no trend expectation, making this exercise obsolete, but it is striking to see that the same method yielded 49 scores of larger mite density in the next week out 92 cases (53.2%; Figure S1). This indicates a clear change in trend, which can be attributed to the impact of the predators on PRM dynamics. This is also confirmed by the observation that PRM densities in the Nordenfors samples in week 6 of the predator treatments were always lower than the PRM densities in any of the samples from the control experiments at the same temperature regime (Figure S1).

The same method of trend scoring can be performed for predator densities in samples taken from the manure (Figure 3, main text). We found 86 scores of larger predator density in the next week out of 92 cases (93.5%). We would certainly expect predator populations to increase in the first few weeks, but their numbers may level off when PRM prey densities decline toward the end of the experiments in week 6 and they are not likely to decline because the predatory mites under test can survive for more than a month when given access to a water source.

*Calculation of sample size required*

Based on the population trend analysis, we have reason to assume that the sampling methods used were good enough to predict qualitatively whether a population increases or decreases. As a quantitative means to estimate population size, however, the sampling methods seem cumbersome, most likely because the sample size was too low. As we had no prior information on the variability of these sampling methods and whether they are representative for the overall population size, there was no way to assess how many samples had to be taken to reliably estimate total population size and we decided to place one Nordenfors trap and to take one litter/manure sample per cage per week. Thus, we used these samples only to test and detect qualitative trends in population size in the time course of the experiments and –most importantly– to relate the samples taken in week 6 to the overall numbers assessed by the method explained in the main text. In this way we were able to assess to what extent the sampling methods were representative for the overall population density of mites and to calculate the sample size (number of cardboard traps) required to estimate population size with given accuracy *δ* (usually 10 to 20%) and a given reliability *α* (usually 5%). Assuming that sample population size is normally distributed and that relative (not absolute) error is what matters, we can calculate sample size *n* from:

*n* ≈ *z(α)^2^ σ2/(δ^2^μ^2^)*

where *z(α)* (≈ 2 for *α*=0.05) is the ordinate on the normal curve corresponding to *α*, *σ^2^* is the population variance and *μ* is the population mean (Cochran, 1977).

Thus, to assess how representative the sampling methods were, we plotted mite densities in the samples of week 6 against the total population size estimated by very intensive sampling in week 6 (Figure S1). The predictive value of the samples is clearly low, as indicated by the values of the correlation coefficients and by the average ranges of PRM numbers in the samples spanning roughly one order of magnitude (i.e. factor 10 between lowest and highest values) (Figure S2). We estimate the standard deviation to be approximately 30.2% of the mean and this implies that the sample size required to estimate population size with reliability α = 0.05 should be 9 (for δ = 0.2) or 36 (for δ = 0.1).

**References**

[Cochran WG](http://en.wikipedia.org/wiki/William_Gemmell_Cochran) (1977) Sampling Techniques (Third ed.). Wiley, New York.

Kirkwood AC (1963) Longevity of the mites *Dermanyssus gallinae* and *Liponyssus sylviarum*. Experimental Parasitology 14:358-366.

Maurer V, Baumgärtner J (1992) Temperature influence on life table statistics of the chicken mite *Dermanyssus gallinae* (Acari: Dermanyssidae). Experimental and Applied Acarology 15:27-40.

Maurer V, Baumgärtner J (1994) A population model for *Dermanyssus gallinae* (Acari, Dermanyssidae). Experimental and Applied Acarology 18: 409-422.

Buffoni G, Di Cola G, Baumgärtner J, Maurer V (1995) A mathematical model for trophic interactions in an acarine predator-prey system. Journal of Biological Systems 3:303-312.

Buffoni G, Di Cola G, Baumgärtner J, Maurer V (1997) The local dynamics of acarine predator-prey (*Cheyletus eruditus* - *Dermanyssus gallinae*) populations: identification of a lumped parameter model. Mitteilungen der Schweizerisches Entomologisches Gesellschaft 70:345-359.

Nordenfors H, Chirico J (2001) Evaluation of a sampling trap for *Dermanyssus gallinae* (Acari: Dermanyssidae). Journal of Economic Entomology 94:1617-1621.

Zenner L, Bon G, Chauve C, Nemoz C, Lubac S (2009) Monitoring of *Dermanyssus gallinae* in free-range poultry farms. Experimental and Applied Acarology 48:157-166.

**Figures Supplementary material**

**Figure S1**

Population trends of poultry red mites (PRM) in Nordenfors-traps (left) and predatory mites in litter/manure (right) in laying hen cages at 3 temperatures (26ºC, 30ºC and 33/25ºC) and for treatments without predators (bold lines), with the predatory mite *Androlaelaps casalis* (thin lines) and with the predatory mite *Stratiolaelaps scimitus* (dashed lines) (2-4 replicates per experiment). Predatory mite densities represent extrapolations from single manure/litter samples to the total number present in and around the manure drawer. PRM inoculated (300 mites) at arrows; 1,000 predatory mites added 12 d (26ºC) or 3 d (higher temperatures) after PRM inoculation.

**Figure S2**

Relation between the number of poultry red mites (PRM) in samples and the total population size estimated from the final (week 6) assessment (x and y axes, both logarithmic). The upper panel concerns samples by a single Nordenfors trap (N-trap) per cage, whereas the lower panel concerns total numbers extrapolated from a single sample from litter and manure in and around the manure tray (multiplication factor = 30). Grey dots, black triangles and black squares concern data obtained at 26ºC, 30ºC and 33/25ºC respectively. The regression line (exponential function shown right) serves the sole purpose of visualizing the overall trend.
