# Supplementary figures and images for: Laboratory tests for controlling poultry red mites (Dermanyssus gallinae) with predatory mites in small ‘laying hen’ cages
Source: Exp Appl Acarol. 2012 Jul 8;58(4):371–83. doi: 10.1007/s10493-012-9596-z (PMC3487000; doi:10.1007/s10493-012-9596-z)

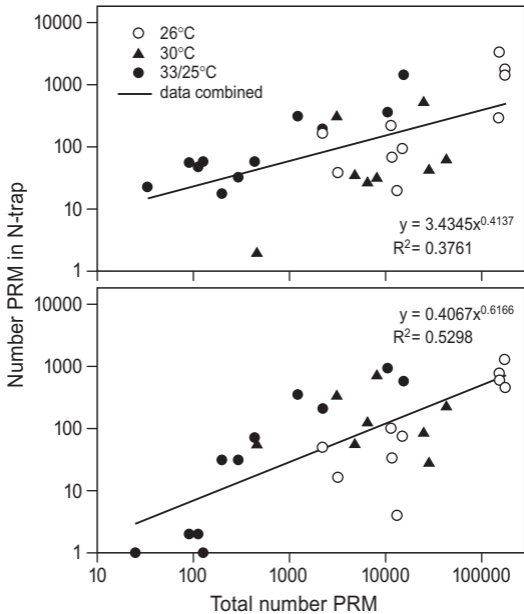

Supplement: Supplementary file 2 — Supplementary material 2 (PDF 44 kb) [file 10493_2012_9596_MOESM2_ESM.pdf]

# Poultry red mites

26°C

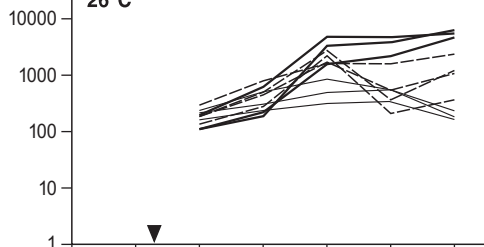

30°C

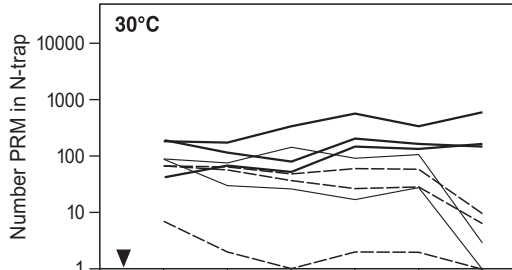

33/25°C

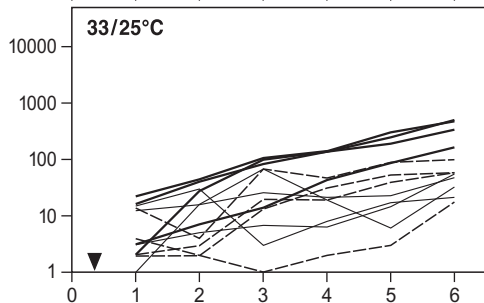

# Predatory mites

26°C

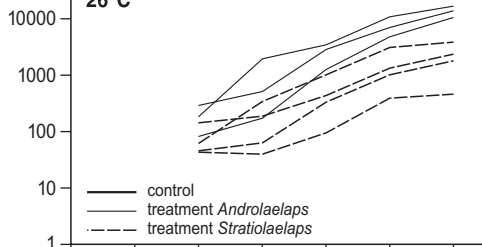

30°C

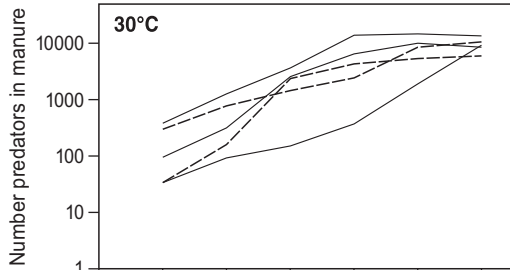

33/25°C

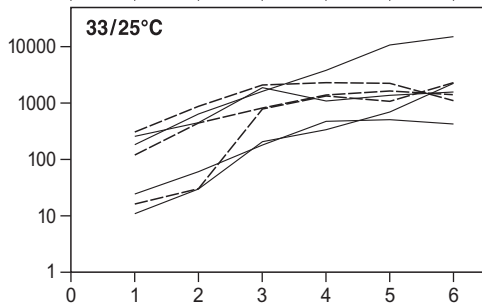

Supplement: Supplementary file 3 — Supplementary material 3 (PDF 70 kb) [file 10493_2012_9596_MOESM3_ESM.pdf]
